# A 6-month follow-up study of the randomized controlled Ma-Pi macrobiotic dietary intervention (MADIAB trial) in type 2 diabetes

**DOI:** 10.1038/nutd.2016.29

**Published:** 2016-08-15

**Authors:** A Soare, R Del Toro, Y M Khazrai, A Di Mauro, S Fallucca, S Angeletti, E Skrami, R Gesuita, D Tuccinardi, S Manfrini, F Fallucca, M Pianesi, P Pozzilli

**Affiliations:** 1 Unit of Endocrinology and Diabetes, Department of Medicine, University Campus Bio-Medico, Rome, Italy; 2 Department of Laboratory Medicine, University Campus Bio-Medico of Rome, Rome, Italy; 3 Center of Epidemiology, Biostatistics and Medical Information Technology, Polytechnic Marche University, Ancona, Italy; 4 Department of Clinical Sciences, La Sapienza University II Faculty, Rome, Italy; 5 International Study Center for Environment, Agriculture, Food, Health and Economics, Tolentino, Italy; 6 Centre for Immunobiology, Barts & The London School of Medicine & Dentistry, Queen Mary University of London, London, UK

## Abstract

**Background:**

In the MADIAB trial (a 21-day randomized, controlled trial in patients with type 2 diabetes (T2D)), intervention with the Ma-Pi 2 macrobiotic diet resulted in significantly greater improvements in metabolic control compared with a standard recommended diet for patients with T2D. We report on a 6-month follow-up study, which investigated, whether these benefits extended beyond the 21-day intensive dietary intervention, in real-world conditions.

**Subjects:**

At the end of the MADIAB trial (baseline of this follow-up study), all participants continued their assigned diet (Ma-Pi or control) for 6 months. The Ma-Pi 2 group followed the Ma-Pi 4 diet during this follow-up study. Forty of the original 51 subjects (78.4%) participated in the follow-up (body mass index, 27–45 kg m^−2^; age, 40–75 years). Primary outcome was percentage change from baseline in HbA1c; secondary outcomes were anthropometric data and lipid panel.

**Results:**

A significantly greater median percentage reduction was observed for HbA1c in the Ma-Pi group (−11.27% (95% confidence interval (CI): −10.17; −12.36)) compared with the control group (−5.88% (95% CI: −3.79; −7.98)) (*P* < 0.001). Total and low-density lipoprotein (LDL) cholesterol increased in both groups with no differences between groups (*P*=0.331 and *P*=0.082, respectively). After correcting for age and gender, the Ma-Pi diet was associated with a higher percentage reduction in HbA1c (95% CI: 2.56; 7.61) and body weight (95% CI: 0.40; 3.99), and a higher percentage increase in LDL cholesterol (95% CI: −1.52; −33.16). However, all participants' total and LDL cholesterol levels remained within recommended ranges (<200 mg dl^−1^ and <100 mg dl^−1^, respectively). The Ma-Pi diet group achieved the target median HbA1c value (<5.7% (39 mmol mol^−1^)) at 6 months.

**Conclusions:**

Both the Ma-Pi and control diets maintained their benefits beyond the 21-day intensive monitored intervention over a 6-month follow-up in real-world conditions. The Ma-Pi diet resulted in greater improvement in glycemic control.

## Introduction

Type 2 diabetes (T2D), obesity and their associated complications and costs are major global public health problems.^1^ The prevalence of T2D has increased worldwide in the past decades: in Italy, approximately 3.6 million people were diagnosed with T2D in 2012 (6.2% of the total population) and it has been estimated that this will increase to 9.0% of the Italian population by 2030.^2^ In 2014, 387 million people worldwide had diabetes and it is predicted that this figure will rise to 592 million by 2035.^1^ Diabetes is currently among the top five causes of death in most high-income countries and resulted in 4.9 million deaths globally in 2014; diabetes is also recognized as a major cause of death in people younger than 60 years.^1^

The increase in the prevalence of T2D, which accounts for approximately 90% of all diabetes cases, is driven by an increase in the numbers of individuals who are overweight or obese.^3^ Indeed, the risk of diabetes increases dramatically as body weight increases; a rise in body mass index from 21 kg m^−2^ (healthy) to 35 kg m^−2^ (obese) can increase the likelihood of developing the disease by a factor of 80.^4^ In line with the increase in the prevalence of diabetes, the number of obese adults, the rates of obesity-related diseases (for example, coronary heart disease, stroke, hypertension and arthritis) and the associated health-care costs are all expected to increase dramatically worldwide over the next 20 years.^4, 5^

In parallel with increased urbanization and economic growth, many countries have experienced drastic changes in food production, processing and distribution, and this has increased the accessibility of unhealthy foods such as highly processed items, high-energy snacks and sugary beverages.^6, 7^ As a result, diabetes prevention and management through lifestyle modification have become increasingly important.

Evidence from prospective observational studies and randomized clinical trials support the importance of individual nutrients, foods and dietary patterns in T2D prevention and management.^8^ The American Diabetes Association's evidence-based position statement on the prevention, therapy and management of T2D recommends a diet that is rich in whole grains, fruits, vegetables, nuts and legumes, and which is low in refined grains and red processed meats.^9^ Although observational and interventional studies spanning two decades provide evidence to support these dietary recommendations,^10, 11^ alternative approaches should be investigated because adherence to currently recommended diets is frequently low.^12^

Macrobiotic diets originally derived from an ancient Eastern philosophy of life; they were updated for Western culture by the Japanese philosopher, Georges Ohsawa,^13^ and further updated by Mario Pianesi who created the five Ma-Pi diets.^14^ The Ma-Pi 2 diet is a low-fat, high-fiber, high-complex carbohydrate, mainly vegetarian diet that was specifically designed for intensive treatment of T2D patients.^14^ As recently demonstrated by the first randomized trial that assessed the effects of the Ma-Pi 2 diet versus the standard nutritional recommendations for T2D (MADIAB trial), patients consuming the Ma-Pi 2 diet during a 21-day dietary intervention in a highly controlled residential setting experienced statistically significant improvements in fasting blood glucose, postprandial blood glucose, glycated hemoglobin (HbA1c), insulin resistance and body weight compared with patients receiving the control diet, even though both diets resulted in improvements in metabolic parameters.^15^

Considerable research has demonstrated that intensive lifestyle interventions^16, 17, 18^ in well-controlled settings are effective for the treatment of T2D, but the positive results achieved with diets in clinical trials are often difficult to replicate in the real world.^19^

For these reasons, the MADIAB trial findings^15^ need to be validated in medium and long-term follow-up studies and the efficacy of this diet in real-life practice needs to be investigated.

We report here the results of a 6-month follow-up study of the MADIAB trial,^15^ which aimed to investigate whether the benefits of the original 21-day intensive dietary interventions extended beyond the original MADIAB trial duration and into everyday life.

## Subjects and methods

### Study design

This 6-month follow-up study involved subjects previously enrolled in the randomized, controlled MADIAB trial, the rationale, design and results of the which have been published previously.^15^ Briefly, the MADIAB trial was a 21-day randomized, controlled open-label study in which participants were randomized in a 1:1 ratio to receive the Ma-Pi 2 macrobiotic diet or a control diet based on dietary recommendations for patients with T2D.^20^ The study enrolled male or female patients aged 40–75 years who were overweight or obese (body mass index, 27–45 kg m^−2^), had received a diagnosis of T2D at least 1 year prior to the start of the trial, and whose disease had been managed with dietary intervention or oral hypoglycemic drugs, or both, for 6 months prior to study entry. Throughout the trial, participants stayed at two different hotels in the same geographic area where dietary compliance was strictly controlled by medical staff.

The follow-up study reported here was designed to assess whether the benefits shown at the end of the MADIAB trial extend beyond the 21-day intensive dietary intervention period. Participating patients were asked to continue their respective assigned diet for 6 months in their own homes with no meals provided by the investigators. This report includes an analysis of data through the 6-month assessment.

The study was conducted in accordance with the Declaration of Helsinki and the Good Clinical Practice guidelines, approved by the Ethics Committee of the University Campus Bio-Medico of Rome (11/13 PAR ComEt CBM) and registered at http://www.ISRCTN.org (ISRCTN10467793). All participants provided written informed consent for participation.

### Subjects

Patients were eligible for inclusion in the follow-up study if they had completed the final visit of the MADIAB trial and gave written informed consent. There were no exclusion criteria. Subjects were recruited by the Endocrinology and Diabetes Unit at University Campus Bio-Medico at the end of MADIAB trial. Subjects were free to discontinue participation at any time.

### Interventions

During the last visit of the MADIAB trial, which coincided with the beginning of this 6-month follow-up study, all participants completing the MADIAB trial were asked to continue for 6 months with either a Ma-Pi macrobiotic diet or the control diet, according to their originally assigned treatment group. For this follow-up study aiming to preserve the significant body weight loss and the improved metabolic control obtained during the 21 days of the trial while keeping patient's compliance, a less restrictive type of MA-PI diet was introduced. For these reasons, the 6-month follow-up intervention diet for the Ma-Pi-assigned patients (who followed a Ma-Pi 2 diet during the MADIAB trial^15^) was the Ma-Pi 4 macrobiotic diet. This is similar to the Ma-Pi 2 diet in terms of the quality and quantity of macronutrients, but includes additional fish-derived protein.

The Ma-Pi 4 diet was specifically designed by Mario Pianesi as a health-promoting diet. It consists mainly of whole grains (brown rice, millet, barley, rye and wheat), vegetables and legumes, but also includes fish, added vegetable oils (extra virgin sesame seeds, rice germ, wheat germ or olive oil), seasonal and local Italian fruit, sesame seeds, nuts (walnuts, hazelnuts, almonds and pine nuts) and fermented products (miso, wandadou jiangyou (soy sauce) and yanzimei (pickled ume plums)). Beicha tea (roasted green tea) and mineral water represent the main source of liquids. Permitted cooking methods are steaming, boiling and roasting.

The control diet in this 6-month follow-up study was the same as used in the MADIAB trial and has been described previously.^15^ Briefly, the control diet was based on the dietary guidelines for T2D recommended by professional societies in Italy,^20^ adapted to the Mediterranean culinary style.

For patients in both dietary groups, energy intake was restricted by limiting calories to 2100 kcal per day and 1900 kcal per day for males and females, respectively, but the two diets differed in nutrient composition. The Ma-Pi 4 diet derived 67% of energy from carbohydrate, 21% from fat and 12% energy from protein, with fiber equal to 27 g 1000 kcal^−1^, while the control diet derived 50% of energy from carbohydrate, 30% from fat and 20% from protein, with fiber equal to 20 g 1000 kcal^−1^. Alcohol consumption was forbidden for both diets.

As previously described,^15^ during the 21-day MADIAB trial, participants attended daily 2-h meetings for nutritional education and cooking instructions conducted by a physician and a registered dietitian and/or a cooking instructor. This was performed to encourage continuation of the respective diets once the trial was completed. Moreover, on the last day of the MADIAB trial, each subject who enrolled in this 6-month follow-up study met for 2 h with a registered dietitian experienced in the use of the assigned diet to establish an appropriate diet plan for the 6-month follow-up. A 10-day menu cycle was devised for both diets and patients were advised to repeat it every 10 days during the 6-month follow-up study. Plans for both diets included five meals per day, with 20% of calories at breakfast, 30% of calories at lunch and 30% of calories at dinner. A snack was included approximately 2.5 h after both breakfast and lunch, with each snack contributing 10% of the total daily calories. Nutritional analysis and menu planning was developed with MètaDieta Software using the Italian Food Composition Tables edited by the National Institute for Food and Nutrition Research (INRAN).^21^ Participants conducted their normal daily routine in their own homes and workplaces, no meals were provided, and dietary adherence and compliance were not controlled during this follow-up study.

Participants were asked not to alter their exercise habits during the 6-month follow-up period. In addition, they were instructed to continue their pre-study oral antidiabetic drug doses without modification throughout the study, unless hypoglycemic symptoms were accompanied by a capillary glucose reading of <70 mg dl^−1^; in such cases, hypoglycemic medications were reduced for participant safety.

### Outcome measurements

The primary outcome of this follow-up study was the percentage change in HbA1c levels from baseline (T0) to the end of the 6-month follow-up (T6) in the Ma-Pi group compared with the control group. Secondary outcomes included percentage change from baseline in body weight and plasma concentrations of total cholesterol, low-density lipoprotein cholesterol (LDLc) and high-density lipoprotein cholesterol (HDLc).

For all participants, venous blood samples were obtained early in the morning after a 12-h fasting period. All biochemical and anthropometric measures were assessed at T0 and T6 by the central laboratory (University Campus Bio-Medico, Rome) and were measured by routine biochemical analysis. Body weight was measured at T0 and T6 before breakfast using a digital scale accurate to 0.1 kg (Seca 700 scale, Seca GmbH, Hamburg, Germany).

### Statistical analyses

A power calculation was performed for the MADIAB trial^15^ but not for the additional 6-month follow-up study reported here. The primary analysis, which was based on the intention-to-treat principle, included all enrolled patients. A non-parametric statistical approach was chosen because the Shapiro test showed that primary outcome and some secondary outcome variables were not normally distributed. Quantitative variables were summarized using percentiles (median and interquartile range). Comparisons between treatment groups were performed using the Wilcoxon rank-sum test and 95% confidence intervals (CIs) for median values. Absolute and percentage frequencies were used for qualitative variables and the Fisher exact test was applied for group comparisons.

The two groups were compared at T0 to determine similarity in terms of demographic characteristics, body weight, lipid levels and carbohydrate metabolic parameters. Percentage differences between values at T0 and T6 were calculated for the primary (HbA1c) and secondary (body weight, total cholesterol, LDLc, HDLc, triglycerides) end points.

**Figure 1:**
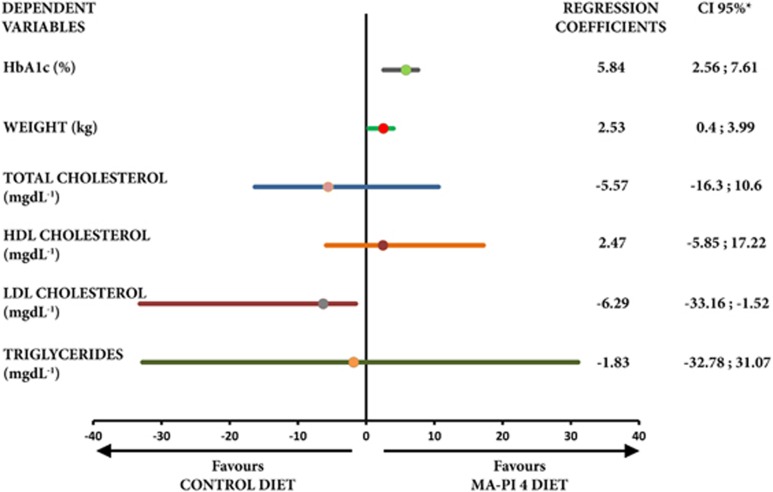
Effect of Ma-Pi diet versus control diet on change in primary and secondary outcomes. The graph shows the effects of the Ma-Pi 4 diet on percentage changes from baseline (T0) to 6 months (T6) in primary and secondary outcomes measures using a multiple quantile regression model adjusted for age and gender. *Coefficients are statistically significant when the 95% CIs do not include zero.

A bivariate analysis was performed to compare changes between the two groups. A linear quantile regression analysis^22^ was performed to estimate the effect of diet (Ma-Pi 4 versus control; explanatory variable) on the median percentage change between T0 and T6 in each measured variable (dependent variable). Each model was adjusted for those variables (gender and age) that could potentially affect the percentage changes in the dependent variables. The results of linear quantile regression analysis were expressed as point and interval estimates of regression coefficients. When the coefficient was positive, the change in the measured variable reduction favored the Ma-Pi 4 diet; when the coefficient was negative, the change favored the control diet. Regression coefficients were considered statistically significant when the 95% CIs did not include zero (Figure 1).

All statistical analyses were performed using R statistical package (Foundation for Statistical Computing, Vienna, Austria) and statistical significance was assessed at a level of probability of 0.05.

## Results

Of the 58 randomized patients in MADIAB, 51 attended the final visit and were classified as having completed the MADIAB trial and being eligible for this follow-up study. A total of 40 subjects (78.4%) agreed to participate and were enrolled in this 6-month follow-up study. The reason given for non-acceptance by all 11 non-participating patients (Ma-PI group, *n*=8; control group, *n*=3) was inability to attend follow-up visits. All the enrolled subjects completed the 6-month follow-up study and were included in the intention-to-treat analysis, with no dropouts.

Baseline demographic and clinical characteristics of patients are presented in Table 1. The only significant difference between groups was LDLc level which was significantly lower in the Ma-Pi 4 group (*P*=0.044) at baseline (T0).

In the bivariate analysis (Table 2), a significant reduction was observed in both groups for the primary outcome, percentage change in HbA1c, over the 6-month follow-up period. The median percentage reduction in HbA1c levels was significantly greater for patients in the Ma-Pi 4 group compared with the control group (*P*<0.001). Furthermore, the Ma-Pi 4 group achieved the median target HbA1c value (<5.7% 39 mmol mol^−1^) at the end of the 6-month follow-up (median values: 5,5% 37 mmol mol^−1^ in the MA-PI group and 6% 42 mmol mol^−1^ in the control group). The target value of <5.7% of HabA1c was chosen in accordance with the ADA recommendations for subjects at risk for diabetes.^9^ There was no significant change in body weight from T0 to T6 in either group, and no difference in body weight change between groups (*P*=0.194). Total cholesterol and LDLc increased significantly in both groups but with no statistical differences between groups (*P*=0.331 and 0.082, respectively). HDLc improved significantly only in the Ma-Pi 4 group (+4.54 (95% CI: 0.65; 8.43)) and triglycerides increased significantly only in the control group (+13.6 (95% CI: 1.84; 25.3)). There were no significant differences between groups for these two variables.

The results of the multiple quantile regression analysis (adjusted for age and gender) are reported in Figure 1. Compared with the control diet, the Ma-Pi 4 diet was associated with a significantly greater percentage reduction in HbA1c level and body weight, and a significantly greater percentage increase in LDLc. Although an increase in median LDLc level was observed in both groups, none of the study participants had LDLc values >100 mg dl^−1^ at the end of the 6-month follow-up. There were no statistically significant differences between groups in percentage change in total cholesterol, HDLc or triglyceride levels.

## Discussion

In this analysis, we assessed glycemic control, body weight and lipid outcomes in a 6-month follow-up of patients who had completed the randomized, controlled MADIAB trial. The original short-term (21-day) MADIAB trial showed that, although both the Ma-Pi and control diets resulted in improvements in metabolic parameters, the Ma-Pi 2 diet was associated with greater reductions in fasting and postprandial plasma glucose, serum cholesterol and body weight than the standard control diet.^15^ In the follow-up study presented here, both the intervention diets further reduced HbA1c from the end of the MADIAB trial but improvements were significantly greater in the Ma-Pi group.

Subjects with T2D in both the Ma-Pi and control groups generally maintained their body weight from T0 to T6. However, after correction for gender and age, a statistically significant reduction in body weight was associated with the Ma-Pi diet. Patients involved in dietary intervention programs frequently have numerous cycles of weight loss and regain.^23^ The fact that both groups retained most of the weight loss achieved during the 21-day MADIAB trial during 6 months of follow-up suggests that the patients' diets did not return to their pre-trial patterns. The greater weight loss in the Ma-Pi group may have been associated with the higher fiber content of this diet (up to 15 g per day more than the control diet). This is supported by the results of a retrospective cohort study of the Canadian population, which found that dietary fiber intake was inversely related to the prevalence of obesity.^23^

Although total cholesterol and LDLc increased significantly in both groups from the end of MADIAB trial and triglyceride levels increased significantly in the control group, the levels remained within the recommended range^9^ for all study subjects (total cholesterol, <200 mg dl^−1^; LDLc, <100 mg dl^−1^; triglycerides, <150 mg dl^−1^). However, after correction for gender and age, the Ma-Pi diet was associated with a significantly greater increase in LDLc than the control diet. In the interpretation of these data, it should be considered that at baseline of this follow-up (which corresponded to the end of the MADIAB trial), the LDLc level was significantly lower in the Ma-Pi group than in the control group (Table 1); this difference was due to the significantly greater reduction in LDLc level obtained in the MA-PI 2 group compared with the control group (−47.9% versus −19.7% *P*<0.001) in the 21-day MADIAB trial.^15^ Moreover, it is of note that the Ma-Pi 4 diet (follow-up study) had a higher fat content than the Ma-Pi 2 diet (21-day MADIAB trial).^15^

The significance of the overall results of this follow-up study is twofold. First, they demonstrate that the improvements obtained in subjects with T2D during an intensive 21-day dietary intervention with a Ma-Pi diet or a recommended standard diet for T2D in a controlled setting^15^ were increased or maintained during 6 months of follow-up, when the patients returned to their normal daily routines and were responsible for their own meal preparation. This is particularly pertinent in the context of T2D management, where low adherence to the currently recommended diets represents one of the main issues with effective lifestyle intervention,^12^ and where the benefits associated with dietary intervention in clinical trials are often difficult to replicate in real-life practice.^19^ Second, our 6-month follow-up is the first medium-term controlled study to assess the beneficial effects of a Ma-Pi macrobiotic diet for T2D. Our study findings confirm that a Ma-Pi diet resulted in significantly greater improvements in glycemic control in patients with T2D than a standard diet recommended for these patients.

Despite clinical recommendations for individuals with diabetes to adopt a healthier lifestyle, adherence to recommended diets is often poor and the majority of patients with T2D fail to control hyperglycemia with diet and exercise alone.^24, 25, 26^ These patients find the lifestyle modification aspect of self-management to be particularly difficult.^27^ Considerable research indicates that barriers to diet are critical influences determining adherence to diet plans.^27, 28^ In a survey of attitudes to adherence to diet and exercise, patients with T2D and their educators indicated that the most important factors influencing adherence to dietary prescriptions include difficulty in maintaining the diet at home or away from controlled settings, and being attracted to foods that are not included in the meal plan.^28^ The patients involved in our 6-month follow-up study were strongly motivated during the 21-day MADIAB trial and participants attended daily 2-h meetings, in their hotels, for nutritional education and cooking instructions conducted by a registered dietitian and/or a cooking instructor.^15^ The aim of these interventions was to encourage continuation of the respective diets once the trial was complete by adapting diet plans and recipes to the patients' everyday life conditions. This intensive training probably helped patients to face potential difficulties in maintaining their respective diets during the 6-month follow-up at home.

Patient perception of treatment efficacy has previously been associated with adherence to that treatment. Pharmacological interventions are typically perceived to be more effective than exercise and diet, and this has resulted in relatively lower adherence to lifestyle modifications.^29^ The great improvements in health status achieved by each subject at the end of the 21-day MADIAB trial^15^ were emphasized by the medical staff during personal meetings with each participant before the beginning of this follow-up study. It is likely that this supportive work was helpful in maintaining a high level of patient confidence in the dietary interventions during the 6-month follow-up, thereby leading to successful medium-term behavioral change.

The results of this study support recommendations for a diet that is rich in fiber, complex carbohydrates, whole grains, vegetables and legumes for patients with T2D. Diets based on these ingredients can achieve good glucose control, decrease insulin requirements and glucose absorption, increase peripheral tissue sensitivity to insulin and control body weight.^30^ Whole-grain fiber has been associated with an improvement in peripheral insulin sensitivity and with increases in pancreatic β-cell secretory capacity.^31, 32, 33^ Moreover, diets rich in whole-grain cereals and legumes are also rich in micronutrients such as magnesium, manganese and zinc; these elements are directly related to improvements in glucose metabolism, insulin sensitivity and β-cell insulin synthesis and secretion, and to prevention of oxidative damage.^34, 35, 36^ In addition, several phenolic compounds from whole-grain cereals have a strong antioxidant capacity *in vivo*. Many of these bioactive compounds are bound to grain cell walls and reach the colon unchanged, only being released during the fermentation process.^37^ Whole-grain cereals are also believed to decrease the postprandial blood glucose response, and to slow gastric emptying and/or delay starch digestion and absorption of starch-derived glucose.^38^ These findings support the recommendation that the majority of dietary carbohydrate should be derived from whole grains to reduce peak blood glucose levels.^38, 39^ Whole-grain cereals and legumes are highly represented in both Ma-Pi 2 and Ma-Pi 4 macrobiotic diets and this may explain, in part, the greater improvements in glycemic control which occurred in the Ma-Pi groups in both the original MADIAB trial and in this follow-up study.

The low-fat, high-fiber Ma-Pi diets^14^ contrast with high-fat, low-fiber diets which have been associated with dysbiosis (imbalance of gut microbiota) and which result in enhanced permeability of the intestinal epithelium and endotoxemia.^40^ Such diets lead to antigen overload of the gastrointestinal immune system and consequent inflammation.^40^ Recent studies have shown a link between dysbiosis, chronic inflammation and several diseases including obesity and obesity-associated metabolic disorders such as T2D ^41, 42^ A *post hoc* analysis of MADIAB trial data demonstrated that the Ma-Pi 2 diet significantly reduced markers of insulin resistance and inflammation.^43^

The success of the control diet in improving metabolic control in T2D should not be overlooked, and is consistent with previous studies on Mediterranean diets.^44, 45^ For example, a Mediterranean diet rich in monounsaturated fatty acids and complex carbohydrates has been associated with lower HbA1c levels and 2-h post-meal glucose levels in patients with T2D.^46^

It should be emphasized that the strength of the MADIAB follow-up study findings are limited by the small sample size. In addition, although nutritionists checked on patients' dietary adherence and general compliance via phone calls throughout the study, no assessment of dietary intake was performed during the 6-month follow-up. The significance of this last issue is twofold. First, this is a study limitation because the degree of adherence to the intervention diet is unknown. However, second, this strategy strengthens the results of the follow-up study because it suggests that the intensive supportive work of the medical staff during the original MADIAB trial allowed the study subjects to incorporate the dietary principles they had learned into everyday settings.

In summary, this 6-month follow-up study of the MADIAB trial in patients with T2D found that both the Ma-Pi 4 macrobiotic diet and a standard diet recommended by professional societies maintained their benefits beyond the 21-day intensive monitored intervention over a 6-month follow-up conducted in ‘real life' settings. However, the Ma-Pi 4 diet resulted in a significantly greater improvement in glycemic control than the control diet, suggesting that the MA-PI diet is a valuable tool in all patients with T2D. Future studies should aim to consider this dietetic approach also in subjects with pre-diabetes and other associated conditions such as hypensulinism and reactive hypoglycemic.

## Figures and Tables

**Table 1:** Baseline characteristics of study participants

*Baseline characteristic*	*Ma-Pi 4 diet*	*Control diet*	P *value*^a^
	*(*n=*17)*	*(*n=*23)*	
*Age and disease history, median (1st; 3rd* *quartiles)*			
Age (years)	65 (61; 73)	64 (59; 70)	0.524
Duration of T2D (years)	7 (2; 12)	4 (2; 11)	0.476
			
*Gender,* n *(%)*			
Male gender	8 (47.1)	12 (52.2)	1.000
			
*Use of OADs,* n *(%)*			
Metformin	10 (58.8)	18 (78.3)	0.296
Other OADs	1 (5.8)	7 (30.4)	0.107
			
*Anthropometric measurements, median (1st; 3rd* *quartiles)*			
BMI (kg m^−2^)	29.3 (28.2; 33.0)	31.1 (27.6; 34.3)	0.538
Body weight (kg)	76.5 (69.9; 89.1)	83.3 (75.2; 99.2)	0.274
			
*Biochemical characteristics, median (1st; 3rd* *quartiles)*			
HbA1c (%)	6.1 (5.9; 6.7)	6.5 (6.2; 6.8)	0.233
HbA1c (mmol mol^−1^)	43 (41; 50)	48 (44; 51)	
Total cholesterol (mg dl^−1^)	121 (112; 138)	154 (133; 181)	0.041
HDL cholesterol (mg dl^−1^)	44 (38; 51)	46 (41; 55)	0.529
Triglycerides (mg dl^−1^)	101 (68; 118)	92 (78; 110)	0.722
LDL cholesterol (mg dl^−1^)	63 (39; 69)	88 (63; 108)	0.044

Abbreviations: BMI, body mass index; LDL, low-density lipoprotein; HDL, high-density lipoprotein; OAD, oral antidiabetic drug; T2D, type 2 diabetes.

a Categorical variables were analyzed with a Fisher exact test; continuous variables were analyzed with a Wilcoxon rank-sum test.

**Table 2:** Percentage changes in primary and secondary outcomes from baseline (T0) to 6 months (T6)

*Outcome*	*Ma-Pi 4 diet*	*Control diet*	P *value*^a^
	*(*n=*17)*	*(*n=*23)*	
	*Median (1st; 3rd* *quartile)(CI 95%)*		
Body weight (kg)	−1.46 (−4.59; 0.78)	0.72 (−2.41; 3.26)	0.194
	(−3.51; 0.60)	(−1.15; 2.59)	
HbA1c (%)	−11.27 (−12.70; −9.84)	−5.88 (−7.90; −1.54)	<0.001
	(−12.36; −10.17)	(−7.98; −3.79)	
Total cholesterol (mg dl^−1^)	21.4 (5.97; 30.00)	14.28 (6.25; 18.00)	0.331
	(12.20; 30.60)	(10.41; 18.15)	
HDL cholesterol (mg dl^−1^)	4.54 (−1.81; 8.30)	5.66 (−0.86; 22.7)	0.388
	(0.65; 8.43)	(−2.12; 13.4)	
Triglycerides (mg dl^−1^)	6.25 (−23.70; 48.50)	13.6 (−2.7; 32.8)	0.652
	(−21.44; 33.94)	(1.84; 25.3)	
LDL cholesterol (mg dl^−1^)	31.37 (17.46; 58.46)	16.66 (0.94; 27.06)	0.082
	(15.66; 47.08)	(8.05; 25.27)	

Abbreviations: CI, confidence interval; LDL, low-density lipoprotein; HDL, high-density lipoprotein.

a *P* values by Wilcoxon rank-sum test refer to percent changes between the two treatment groups.
